# A Heptaplex PCR Assay for Molecular Traceability of Species Origin With High Efficiency and Practicality in Both Raw and Heat Processing Meat Materials

**DOI:** 10.3389/fnut.2022.890537

**Published:** 2022-06-23

**Authors:** Song Zhou, Guowei Zhong, Hanxiao Zhou, Xiaoxia Zhang, Xiaoqun Zeng, Zhen Wu, Daodong Pan, Jun He, Zhendong Cai, Qianqian Liu

**Affiliations:** ^1^Key Laboratory of Animal Protein Deep Processing Technology of Zhejiang Province, College of Food and Pharmaceutical Sciences, Ningbo University, Ningbo, China; ^2^Center for Global Health, School of Public Health, Nanjing Medical University, Nanjing, China; ^3^Ordos Agriculture and Animal Husbandry Technology Extension Centre, Ordos, China; ^4^Institute of Environmental Research at Greater Bay Area, Guangzhou University, Guangzhou, China

**Keywords:** heptaplex PCR, adulteration, meat species, mitochondrial sequence, commercial foodstuffs

## Abstract

Frequent meat frauds have become a global issue because adulteration risks the food safety, breaches market rules, and even threatens public health. Multiplex PCR is considered to be a simple, fast, and inexpensive technique that can be applied for the identification of meat products in food industries. However, relatively less is known about a multiplex PCR method authenticating seven animal species simultaneously in one reaction due to technological challenge. Through screening new species-specific primers and optimizing PCR system, a heptaplex PCR method was established, which could simultaneously detect seven meat ingredients of camel (128 bp), pigeon (157 bp), chicken (220 bp), duck (272 bp), horse (314 bp), beef (434 bp), and pork (502 bp) in a single-tube reaction. DNA sequencing solidly validated that each set of primers specifically amplified target species from total DNA mixtures of seven meat species. The developed multiplex assay was stable and sensitive enough to detect 0.01–0.025 ng DNA from various meat treatments including raw, boiled, and autoclaved meat samples or target meat content of 0.1% total meat weight, suggesting the suitability of the heptaplex PCR technique for tracing target meats with high accuracy and precision. Most importantly, a market survey validated the availability of this multiplex PCR technique in real-world meat products with a good application foreground.

## Introduction

Frequent meat frauds have become a global issue, which perhaps risks the food safety, breaks the rules of the market, and even threatens public health (1, 2). Specifically, since the European horsemeat scandal in 2013, the events regarding ingredient substitution of expensive meat with low-cost ones for extra economic benefits have frequently occurred worldwide (3). The false information on the label will mislead the choices of consumers, resulting in serious religious issues and health problems (4, 5). For example, meat products harboring pork ingredients are not permitted in Islamic countries (6, 7). Similar to soy allergy, certain meat species may trigger allergic reactions, especially for sensitized patients, which may cause a severe health risk of infectious diseases, metabolic disorders, and allergies (8–11). Therefore, a practical technique to identify the animal origin with rapid, sensitive, and accurate characteristics is of great importance (10, 11).

The techniques have been continuously evolved for identifying the origin of meat species in the recent years (2, 12–15). DNA molecules present in every cell and possess high stability, and therefore, DNA-based techniques combined with polymerase chain reaction (PCR) provide more reliable methods in discriminating meat species (13, 16, 17). Both conventional multiplex and real-time PCR techniques are considered to be reliable methods with high sensitivity and specificity in authenticating meat species (18). Real-time PCR techniques are generally used for quantifying the amount of a target sequence in a reaction system. As reported, accurate quantification could only be achieved with a proper reference material, as some ingredients in the recipe might be co-extracted with the DNA and interfere with the quantification process (19, 20). In this regard, multiplex PCR presents a simple, efficient, and inexpensive technique, which are being widely applied for qualitatively authenticating animal origin in food industries (1). Nowadays, this technique remains to be considered as a practical method used for identifying the origin of meat species.

Mitochondrial DNA has high copy number in each cell and possesses strong stability, which has allowed a low detection limit and broad availability in various meat products (21). Extensive studies have found that mitochondrial genes such as cytochrome *b*, 12S and 16S rRNA, D-loop, ATPase subunits 6 and 8, and NADH dehydrogenase have been broadly targeted for PCR protocols in the identification of meat species (19, 21–24). Here, using mitochondrial DNA sequences retrieved from camel, pigeon, chicken, duck, horse, beef, and pork, seven sets of primers that specifically amplified seven animal species were designed with differential lengths through PCR assays. Simplex and multiplex PCR assays demonstrated the specific, sensitive, and efficient properties of all primers. This study ultimately constructs a heptaplex PCR method, which can simultaneously detect seven ingredients in meat products.

## Materials and Methods

### DNA Extraction

Fresh pure meat of camel, pigeon, chicken, duck, horse, beef, and pork was obtained from local retailers and markets, which was transported on ice to the laboratory for processing immediately. A total of 67 raw or heat processing of meat balls (10), meat slices (7), kebab (5), sausages (10), cutlets (3), drysaltery (7), jerky (10), steak (4), dry meat stripe (5), braised pigeon (3), and fried pigeon (3) were obtained from markets as well as from online supermarket platform. All samples were stored at −80°C to inhibit DNA degradation. Genomic DNA from meat samples was isolated using the EasyPure^®^ Genomic DNA Kit (Beijing Trans Gen Biotech Co., Ltd., Beijing, China) according to the manufacturer's instructions. DNA concentration was examined by a NanoDrop 2000 spectrophotometer (NanoDrop 2000, UV–Vis spectrophotometer, USA) (25).

### Design of Seven Species-Specific Primer Pairs

Mitochondrial genes were selected as targets for designing primers according to high divergence and conservation within the animal species (23). As seen in Table 1, sequences of cytochrome c oxidase subunit III of camel (GenBank accession no. MH109991.1), NADH dehydrogenase subunit 6 of pigeon (KP168712.1), D-loop of chicken (MK163565.1), ATPase subunit 6 of duck (MK770342.1), NADH dehydrogenase subunit 4 of horse (MN187574.1), cytochrome c oxidase subunit II of beef (MN714195.1), and 16S rRNA gene of pork (KJ746666.1) were retrieved from the National Center of Biotechnology Information (NCBI) database. The conservative and variable regions among animal species were examined by MEGA6 alignment tool. Combined Oligo 7.0 with BLAST programs (www.ncbi.nlm.nih.gov/blast/), new primers were designed according to physical parameters of melting temperature, cross-reactivity, self-complementarity, and secondary structures (7). Primers were synthesized by Shanghai Sangon Biological Engineering Technology & Services Co., Ltd. (Shanghai, China). To detect the mismatch present in the target and non-target species, all primer pairs were *in silico* screened against 14 land animals, camel (*Camelus bactrianus*), pigeon (*Columba livia*), chicken (*Gallus gallus*), duck (*Anas platyrhynchos*), horse (*Equus caballus*), cattle (*Bos taurus*), pig (*Sus scrofa*), turkey (*Meleagris gallopavo*), goose (*Anser cygnoides*), sheep (*Ovis aries*), ostrich (*Struthio camelus*), dog (*Canis lupus*), rabbit (*Oryctolagus cuniculus*), cat (*Felis catus*), and 3 aquatic species, small yellow croaker (*Larimichthys polyactis*), black carp (*Mylopharyngodon piceus*), and tuna (*Thunnus orientalis*) using ClustalW software. Specificity of primer pairs was examined by simplex PCR assays, respectively.

**Table 1:** Oligonucleotide primers for meat species used in this study.

**Primers**	**Genes**	**Sequence (5'- 3' direction)**	**Amplicons (bp)**	**Reference or source**
Pork	16S rRNA	GAAGCCTTTCTCCTCGCACAC	502	this study
		CCCAACCGAAATTGCTAGTCCA		
Beef	Cytochrome c oxidase subunit II	GCTGACCCATACAAGCACGA	434	this study
		CGTAATATAAGCCTGGACGGGAC		
Horse	NADH dehydrogenase subunit 4	TAGAAGCCCCAATTGCCGGAT	314	this study
		TATTGATGATGTAAGGCCGTGAG		
Duck	ATPase subunit 6	TCCCAGCCCTATTGTTCCCAT	272	this study
		TGTTAGTAGGGTAGCAAGCCACA		
Chicken	D-loop	CCCTACTTGCCTTCCACCGTA	220	this study
		CTTGAATAGCACTCCGCACCC		
Pigeon	NADH dehydrogenase subunit 6	CACCGCCCGAATCGCACCAC	157	this study
		AGGGATGTTTTCTGTCCGGTT		
Camel	Cytochrome c oxidase subunit III	GCTCCACTTTCCTAACCGTGT	128	this study
		ATAGAGGAACAGCCAGACGACA		
Eukaryotes	12S rRNA	CAACTGGGATTAGATACCCCACTAT	456	(29)
		GAGGGTGACGGGCGGTGTGT		
Eukaryotes	16S rRNA	AAGACGAGAAGACCCTATGGA	240	(28)
		GATTGCGCTGTTATCCCTAGGGTA		
Eukaryotes	18S rRNA	AGGATCCATTGGAGGGCAAGT	99	(30)
		TCCAACTACGAGCTTTTTAACTGCA		

### Simplex and Multiplex PCR Assays

Polymerase chain reaction assays were performed as previously described (25). For simplex PCR, PCR amplification was achieved using EasyTaq^®^ DNA Polymerase Kit (Beijing TransGen Biotech Co., Ltd., Beijing, China). PCR system was composed of 2.5 μl of 10 x EasyTaq^®^ Buffer, 2 μl of 2.5 mM dNTPs, 0.5 μl of EasyTaq DNA polymerase, 0.5 μl of 10 μm each primer, genomic DNA of each species, and refilled ddH_2_O to 25 μl in a single reaction. The reaction was elicited by a 5-min denaturation at 94°C, followed by 34 cycles (94°C for 30 s, 63°C for 30 s, and 72°C for 45 s) and a final elongation at 72°C for 5 min. Using the same PCR amplification condition as that of simplex PCR, we employed a set of seven species-specific primers and corresponding genomic DNA as templates to develop a heptaplex PCR assay. For multiplex PCR, PCR system included 2.5 μl EasyTaq^®^ Buffer (10 x), 2 μl dNTPs (2.5 mm), 0.5–1 μl EasyTaq DNA Polymerase (5 units μl^−1^), 0.5 μl each primer of seven species (10 μM), 1 μl genomic DNA of each species at the indicated concentrations from 10 to 0.1 ng μl^−1^, and refilled ddH_2_O to 25 μl. All PCR fragments were amplified using T100™ Thermal Cycler (Bio-Rad, Germany) and analyzed through 4% agarose gels using 4S GelRed Nucleic Acid Stain, which were visualized by Gel Doc^TM^ XR+ System with Image Lab^TM^ Software (BIO-RAD) (26).

### Sequencing of PCR Products

Each PCR product was purified from the gel with DiaSpin DNA Gel Extraction Kit (Shanghai Sangon Biological Engineering Technology & Services Co., Ltd., Shanghai, China) and cloned into a *pEASY*^®^-T5 Zero Cloning vector (TransGen Biotech Co., Ltd., Beijing, China) according to the manufacturer's protocol. Each plasmid DNA was extracted using a SanPrep Column Plasmid Mini-Preps Kit (Shanghai Sangon Biological Engineering Technology & Services Co., Ltd., Shanghai, China). PCR amplification with vector primers M13F (5'-GTAAAACGACGGCCAGT-3') and M13R (5'- CAGGAAACAGCTATGAC-3') was carried out using the template of plasmid DNA and then sequenced by an automated DNA sequencer (Applied Biosystems, Foster City, CA, USA). Determination of DNA base composition of the sequences was accomplished by a BLAST search against the NCBI nucleotide database.

### Evaluation of Primers' Specificity, Sensitivity, and Reproducibility

The specificity of each primer pair was assessed by using template DNA extracted from all species (camel, pigeon, chicken, duck, horse, cattle, pork, turkey, goose, sheep, rabbit, ostrich, dog, cat, small yellow croaker, tuna, and black carp). For the preliminary phase of the experiment, simplex and multiplex PCR assays were individually performed using the template DNA isolated from raw animal species. For sensitivity test, a series of PCR assays were performed using serial dilutions of the premixed genomic DNA templates of all target species in one reaction. A total of ten concentrations of the target templates ranging from 10 ng to 0.01 ng were used for PCR amplification. To further determine the sensitivity of model mixtures, each raw meat tissue of pork, horse, duck, chicken, camel, and pigeon was weighed at 0.1, 0.25, 0.5, 1, 2.5, 5, 10, and 15 of total weight, respectively. Then, all meat weighed at the same proportion were gathered with beef together and then homogenized separately using different triturators to avoid cross-contaminations. The dynamic range and the limit of detection were determined through 4% agarose gel analysis. For reproducibility assay, raw meat samples were boiled (97–99°C, 30 min) and microwave-cooked (750 W, 10 min) and then used for DNA isolation. Similar to sensitivity test, PCR amplification was carried out and the reproducibility was analyzed by agarose gel analysis (27).

## Results

### Specificity Assays of the Designed Primers

**Figure 1:**
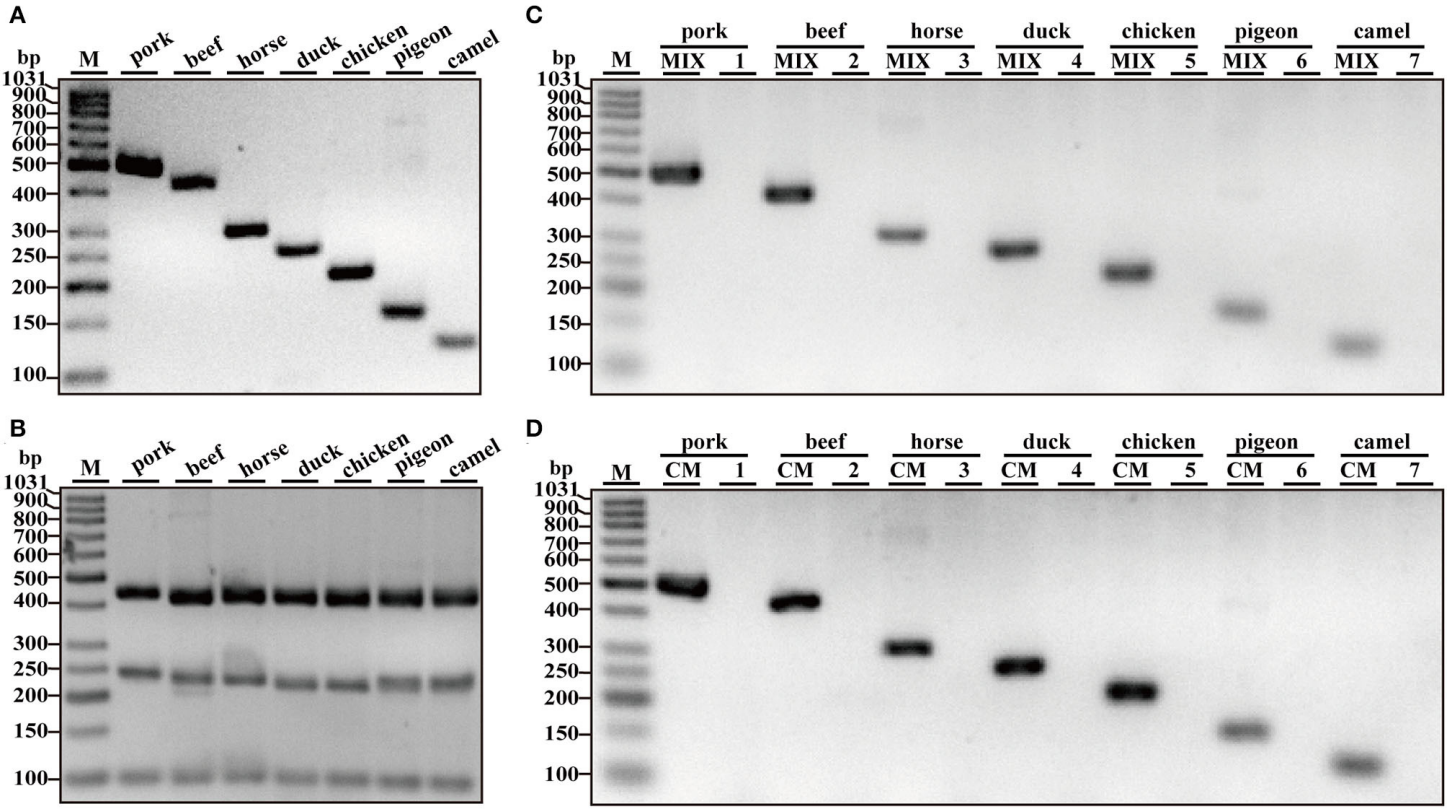
Verification of primer specificity with conventional simplex PCR. **(A)** Simplex PCR detection using species-specific primers for camel, pigeon, chicken, duck, horse, beef, and pork origin and respective genomic DNA as a template. **(B)** PCR amplification with premixed universal primers of eukaryotic 12S rRNA, 16S rRNA, and 18S rRNA genes for each meat species, respectively. **(C)** PCR amplification using individual template DNA from camel, pigeon, chicken, duck, horse, beef, and pork species. MIX, a mixture of seven primer pairs of camel, pigeon, chicken, duck, horse, beef, and pork species; 1–7, a mixture of six primer pairs of six nontarget species. **(D)** PCR amplification with species-specific primers for camel, pigeon, chicken, duck, horse, beef, and pork species. CM, a complete mixture of seven species including camel, pigeon, chicken, duck, horse, beef, and pork; 1–7, a complete DNA mixture of six meat species except target species. Lane M is ladder DNA.

To obtain species-specific primers, many candidate primers for each species were checked. Using the primer pairs in Table 1, PCR amplification produced the only band with target meat species but not non-target species after the analysis of gel electrophoresis. As shown in Figure 1A, PCR products showed differential bands with the predicted size of 128, 157, 220, 272, 314, 434, and 502 bp for camel, pigeon, chicken, duck, horse, beef, and pork species, respectively. To ensure the quality of genomic DNA templates, three sets of universal eukaryotic primers were selected as positive controls in a single PCR, which targets 18S rRNA, 16S rRNA, and 12S rRNA genes with the predicted size of 99-bp, 240-bp, and 456-bp PCR fragments in all meat species (28–30). In our study, all meat samples generated the target PCR bands with intensities similar to each other (Figure 1B), suggesting the good quality of genomic DNA existing in meat resource. Using genomic DNA of a single meat species as the template, PCR product was obtained just using all primer mixtures of seven meat species, but not that of six non-target primer pairs without target counterpart (Figure 1C). In addition, species-specific primer pair generated PCR fragments with template DNA mixture of seven but not six meat species excluding target one through PCR amplification (Figure 1D), suggesting that the primer pair can specifically amplify target species. To make the conclusion more solid, the 128, 157, 220, 272, 314, 434, and 502-bp amplicons in Figure 1D were cloned and then sequenced. As expected, DNA sequencing validated the accurate amplification of camel, pigeon, chicken, duck, horse, cattle, and pork by a BLAST search against the NCBI nucleotide database. In addition, the specificity of all primer pairs was validated against 17 animal species which indicated through PCR analysis (data not shown). Collectively, this assay demonstrates that new primers are highly specific and are suitable for the authentication of meat species in real-world foodstuffs.

### Sensitivity Assays of Heptaplex PCR

**Figure 2:**
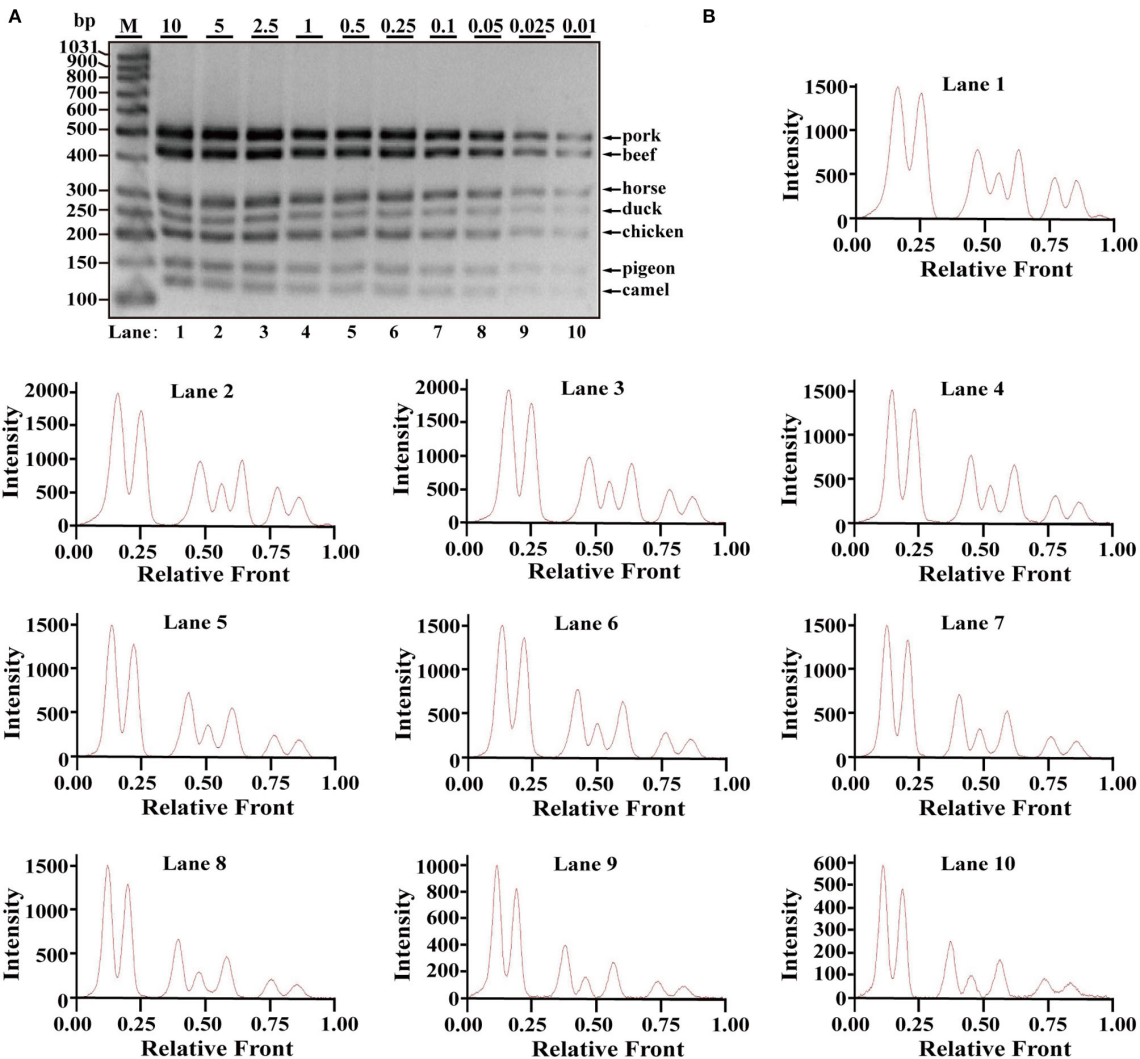
Validation of the sensitivity of multiplex PCR assay. **(A)** Gel image of PCR fragments amplified by multiplex PCR using premixed DNA templates of seven species (10, 5, 2.5, 1, 0.5, 0.25, 0.1, 0.05, 0.025, and 0.01 ng) with species-specific primers of seven meat species in a single PCR. **(B)** The corresponding electropherogram of gel image represented pork, beef, horse, duck, chicken, pigeon, and camel in each lane. Lanes 1–10 are presented with labels (10, 5, 2.5, 1, 0.5, 0.25, 0.1, 0.05, 0.025, and 0.01) in **(A)**. The value of number at the horizontal line means the relative position of peaks distant from the top of agarose gel. The value of number at the vertical line means the fluorescent intensity of DNA-bound dyes using 4S GelRed nucleic acid stain. Lane M is ladder DNA.

After the specificity analysis of each set of primers, a heptaplex PCR system was developed using seven pairs of species-specific primers. To analyze the dynamic range as well as the limit of detection (LOD), multiplex PCR assays were performed with serial dilutions of template DNA of each target species ranging from 10 ng to 0.01 ng in per PCR. As shown in Figure 2A, top bands of five meat species (pork, beef, horse, duck, and chicken) were obviously observed at all indicated DNA concentrations ranging from 0.01 to10 ng DNA. In comparison, pigeon and camel had relatively weak bands under the condition of 0.01 and 0.25 ng template DNA, especially 0.01 ng DNA; however, they seemed to be recognized.

**Figure 3:**
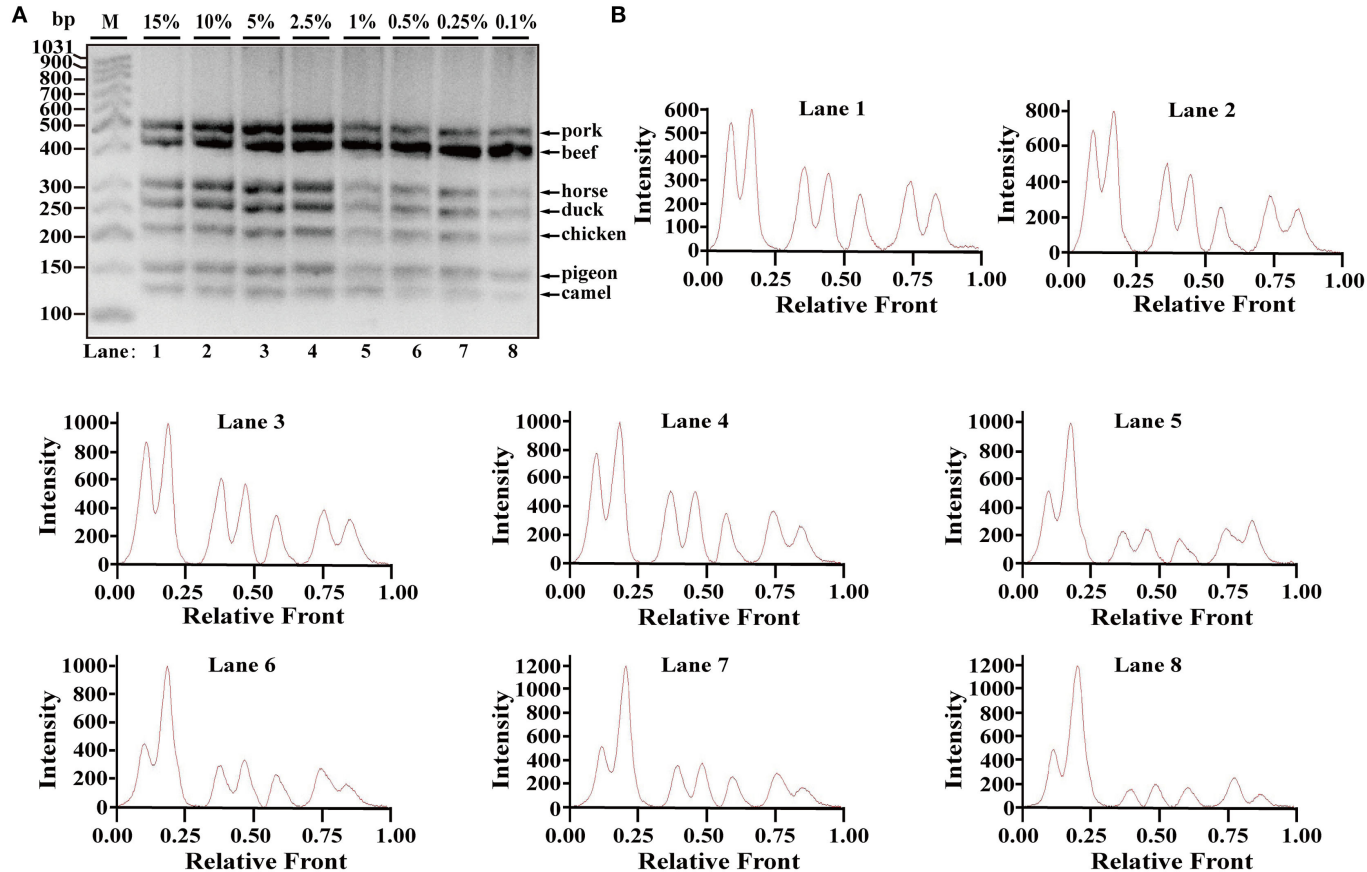
Validation of the sensitivity of multiplex PCR assay. **(A)** Gel image of PCR fragments amplified by multiplex PCR using model mixtures of pork, horse, duck, chicken, pigeon, and camel added to beef at 15, 10, 5, 2.5, 1, 0.5, 0.25, and 0.1% of total weight with species-specific primers of seven meat species in a single PCR. **(B)** The corresponding electropherogram of gel image represented pork, beef, horse, duck, chicken, pigeon, and camel in each lane. Lanes 1–8 are presented with labels (15, 10, 5, 2.5, 1, 0.5, 0.25, and 0.1%) in **(A)**. The value of number at the horizontal line means the relative position of peaks distant from the top of agarose gel. The value of number at the vertical line means the fluorescent intensity of DNA-bound dyes using 4S GelRed nucleic acid stain. Lane M is ladder DNA.

Using Image Lab^TM^ Software, the electropherograms were drawn based on the bands. The visible bands were matched with intact peak patterns, whereas weak bands were equipped with defective peak patterns. As can be seen from Figure 2B, the fluorescent intensities were gradually decreased from lanes 1 to 10 along with reduced content of genomic DNA template, reflecting their reduced PCR products. In accordance with gel view, electropherograms showed intact peak patterns of pork, beef, horse, duck, and chicken in lines 1–10. By contrast, lower intensities of camel and pigeon were found in all lines, whereas defective peak pattern of both camel and pigeon was present in line 10. Collectively, it was postulated that LOD of heptaplex PCR method was 0.01 ng DNA for pork, beef, horse, duck, and chicken, whereas it was 0.025 ng DNA for pigeon and camel. In addition, model beef adulterated with six meat tissues of pork, horse, duck, chicken, camel, and pigeon at 0.1, 0.25, 0.5, 1, 2.5, 5, 10, or 15% of total weight was individually used for genomic DNA extraction. Using the multiplex PCR method, the specific amplicons for each species were clearly displayed even at target meat percentages of 0.1% (Figures 3A,B).

### Reproducibility Assay of Heptaplex PCR in Heat Processing Meat

To examine the efficiency of each set of primers for detecting thermally processed meat, both boiled and microwave-cooked treatments were adopted to process raw meat samples. After genomic DNA isolated from boiled meat samples, heptaplex PCR was carried out and the products were analyzed through 4% agarose gels. As shown in Supplementary Figures S1A, S1B, seven bands of seven meat species were obviously observed at the range of 0.01–10 ng DNA, and intact peak patterns of seven species were found in lines 1–10, suggesting that LOD of heptaplex PCR method was 0.01 ng DNA for pork, beef, horse, duck, chicken, pigeon, and camel under the condition of boiled treatment. Using genomic DNA isolated from microwave-cooked meat samples, top bands of five meat species (pork, beef, horse, duck, and chicken) were obviously observed at the range of 0.025–10 ng DNA, and intact peak patterns of pork, beef, horse, duck, and chicken were found in lines 1–9 (Supplementary Figures S2A, S2B). Combined the data obtained from both boiled and microwave-cooked treatments, the threshold value for discrimination of heat processing meat was about 0.01–0.025 ng DNA. The results suggest that new primers are suitable for authenticating animal species in real-world meat products.

### Application of Multiplex PCR Assays on Commercial Foodstuffs

Typical cases of intentional meat adulteration involve the substitution or addition of animal ingredients not declared on the label of the products. A total of 67 commercial samples of pork, beef, horse, pigeon, and camel were purchased for the identification of real-world meat products through this heptaplex PCR technique. As summarized in Table 2, most of meat samples contained the identical ingredients as labeled without any contamination. However, some samples that declared to be 100% pure meat content were found to be substituted with other meat ingredients. As illustrated, 5 of 15 (33.3%) pork samples, 6 of 15 (40.0%) beef samples, 3 of 12 (25%) horse samples, 1 of 10 (10%) pigeon samples, and 3 of 15 (20%) camel samples contained some meat ingredients unlisted. This survey unmasked that cheap meat of chicken, duck, and pork are likely to frequently used as a substitute ingredient for red meat. Most importantly, the survey further validated the efficiency of heptaplex PCR technique in authenticating commonly consumed meat species.

**Table 2:** Results of multiplex PCR assay performed on commercial meat products.

**Products**	**Number**	**Labeled**	**Detected species**							**Adulteration**
			**Pork**	**Beef**	**Horse**	**Duck**	**Chicken**	**Pigeon**	**Camel**	
**Pork**	15									5(33.3%)
Meat balls	5	pork	5/5	—	—	1/5^a^,1/5^b^	1/5^a^	—	—	
Sausages	5	pork	5/5	—	—	1/5^a^,1/5^b^	1/5^a^,1/5^b^	—	—	
Drysaltery	2	pork	2/2	—	—	—	—	—	—	
Cutlets	3	pork	3/3	—	—	—	1/3	—	—	
**Beef**	15									6(40%)
Meat balls	5	beef	1/5^a^	5/5	1/5^b^	1/5^c^	1/5^c^	—	—	
Meat slices	5	beef	1/5^a^	5/5	1/5^b^	—	1/5^a^	—	—	
Kebab	5	beef	1/5^a^	5/5	—	—	—	—	—	
**Horse**	12									3(25%)
Meat slices	2	horse	—	—	2/2	—	—	—	—	
Sausages	5	horse	1/5^a^	—	5/5	1/5^a^	1/5^b^	—	—	
Jerky	5	horse	1/5	—	5/5	—	—	—	—	
**Pigeon**	10									1(10%)
Steak	4	pigeon	—	—	—	—	1/4	3/4	—	
Braised pigeon	3	pigeon	—	—	—	—	—	3/3	—	
Fried pigeon	3	pigeon	—	—	—	—	—	3/3	—	
**Camel**	15									3(20%)
Drysaltery	5	camel	1/5	—	—	—	—	—	5/5	
Dry meat stripe	5	camel	1/5	—	—	—	—	—	5/5	
Jerky	5	camel	—	—	1/5	—	—	—	5/5	

*In each row, the meat samples labeled with same letter (a or b) represent the identical meat samples, whereas different letters indicate a difference in meat samples*.

## Discussion

Food frauds are the topical issues that must be addressed due to quality and safety purposes and to maintain consumers' trust (31). Nowadays, adulteration practice has been ingeniously done with the morphological and physical characteristics similar to pure meat (6). Multiplex PCR assays have some advantages in discriminating animal origins, because they are easily accomplished through simple agarose gel analysis and dramatically minimizes the cost (32–34). As shown in Supplementary Table S1, much information is available on recently published multiplex PCR methods. While the detection number of meat origin mainly focuses on two to six species in one reaction, relatively less is known on the identification of more meat species in a single PCR. Although some studies have authenticated more than ten animal species, they are achieved by two-tube multiplex PCR assays (7, 27). Accordingly, it is still lack of reliable, low-cost, and high-throughput detection methods for supervising more species origin of meat. This study has set up a multiplex PCR method for simultaneous identification of seven meat species including ruminant, poultry, and pork materials.

Due to the high homology existing in meat species, proper target genes are required for establishing the multiple PCR system. Mitochondrial DNA sequence has variable region with intraspecific and interspecific polymorphism in animal cells, which is highly suitable for the discrimination of closely related animal species (35). Besides, mitochondrial DNA sequences have multiple copies within the ring structure, which is more stable during meat processing. Therefore, the polymorphism site of mitochondrial sequences was selected as a target region for designing species-specific primers in meat source. Because multiple targets can be simultaneously detected by a multiplex PCR method in a single/assay platform, species-specific primers should be analyzed, screened, and optimized to eliminate interaction among animal species and thus ruled out the cross-reactivity in meat resource. Based on this, many candidates of species-specific primers were designed throughout target mitochondrial DNA sequences and ultimately determined seven sets of species-specific primers for camel (128 bp), pigeon (157 bp), chicken (220 bp), duck (272 bp), horse (314 bp), beef (434 bp), and pork (502 bp). The specificity test validates that all primers are specific to each own species without cross-reactivity with other 16 animal species indicated. In addition, DNA sequencing further validates the specificity of species-specific primers. Notably, different lengths of multiple amplicons as well as the competition among PCR amplification modules may result in different PCR efficiencies and, consequently, affect LOD of multiplex PCR methods. As reported, the detection limits of multiplex PCR assays vary from 1 pg to 0.32 ng (Supplementary Table S1). Here, LOD of this multiplex PCR technique is about 0.01–0.025 ng DNA in various meat samples of raw, boiled, and microwave-cooked meat. To further evaluate the availability of multiplex PCR in real-world foodstuffs, model mixtures were constructed by adding each meat sample of pork, horse, duck, chicken, camel, and pigeon weighed at 0.1 to 15% of total weight to beef. The proposed multiplex PCR method can clearly detect even at target meat percentages of 0.1% (Figure 3), whereas target meat content can be availably detected at percentages of 0.01 to 9.1% total weight (Supplementary Table S1). The data suggest that this method is adequate for meat authentication.

The demand for beef is increasing worldwide due its nutritional value of protein, amino acids, and trace elements. A high incidence of beef substitution and mislabeling is prevailing for monetary benefits. Hence, detection of beef adulteration is indispensable for safeguarding consumer rights and food safety. Application of multiplex PCR assay on commercial meat products revealed some interesting and shocking findings that cheap poultry and pork meats are frequently adulterated to meat products (Table 2). In accordance with previous studies, meat resource is frequently adulterated with cheap- or poor-quality meat such as chicken, duck, and pork, which easily escapes visual detection (36–38). Perhaps, economic benefits are a critical factor for the substitution of expensive and high-quality meat with inferior and low-cost ones, and therefore, the amounts of adulterated ingredients should be easily detected. In this regard, these traces such as 0.1% adulterant may be mixed unintentionally with other origins from either the place of origin or at processing level. Identification of animal origin can ensure the authenticity and traceability of meat products, protecting consumers' health and complying with religious faith. However, serially diluted DNA from raw meats could not be used as a reference for DNA isolated from commercial meat samples, as DNA co-extraction might occur due to the presence of inclusion in the recipe and interferes with the quantification process. Therefore, multiplex PCR assays perhaps fail to determine the percentage of adulteration of a real-world meat product. In addition, less is known about how the magnitude of adulteration poses health threats, especially for sensitized patients. It is necessary to deal with the challenging issues in the future study. Nonetheless, this study provides a developed heptaplex PCR assay showing a reliable, efficient, and sensitive detection method for the discrimination of meat species origin in actual adulteration event.

## Conclusions

This study provides a heptaplex PCR technique for the identification of meat products mislabeling prevailing in real-world foodstuffs, which achieves simultaneous detection of seven animal species of camel, pigeon, chicken, duck, horse, beef, and pork species. Meat origin can be availably detected at the concentration of 0.01–0.025 ng DNA or target meat content of 0.1% total meat weight in one-tube reaction system, indicating that this assay has qualified for the authentication of species origin of meat in real-world foodstuffs. The availability of the assay has been further confirmed by the findings obtained from application of multiplex PCR on commercial meat products. Collectively, this multiplex PCR technique could be more broadly used for the identification of species origin of meat in foodstuffs through the analysis of simple agarose gel.

## Data Availability Statement

The original contributions presented in the study are included in the article/Supplementary Material, further inquiries can be directed to the corresponding authors.

## Author Contributions

DP, ZC, and JH: conception and design of the investigation and work. SZ, GZ, HZ, XZh, XZe, and ZW: completion of the experiments. SZ, GZ, ZC, JH, QL, and DP: evaluation and analysis of the results. SZ, GZ, ZC, and JH: manuscript writing. SZ, GZ, HZ XZh, XZe, ZW, DP, JH, QL, and ZC: final approval of manuscript. All authors contributed to the article and approved the submitted version.

## Funding

This work was financially supported by the National Natural Science Foundation of China (NSFC) (31901668), the Natural Science Foundation of Zhejiang Province of China (LY22C200002), the Scientific Research Fund of Zhejiang Provincial Education Department (Y201940932), the Natural Science Foundation of Ningbo (2019A610436 and 2021J108), and the School Research Project in Ningbo University (XYL19011).

## Conflict of Interest

The authors declare that the research was conducted in the absence of any commercial or financial relationships that could be construed as a potential conflict of interest.

## Publisher's Note

All claims expressed in this article are solely those of the authors and do not necessarily represent those of their affiliated organizations, or those of the publisher, the editors and the reviewers. Any product that may be evaluated in this article, or claim that may be made by its manufacturer, is not guaranteed or endorsed by the publisher.
