# Supplementary material for: A Heptaplex PCR Assay for Molecular Traceability of Species Origin With High Efficiency and Practicality in Both Raw and Heat Processing Meat Materials
Source: Front Nutr. 2022 Jun 23;9:890537. doi: 10.3389/fnut.2022.890537 (PMC9260169; doi:10.3389/fnut.2022.890537)
Supplement: Supplementary file 1 [file Data_Sheet_1.PDF]

A heptaplex PCR assay for molecular traceability of species origin with high efficiency and practicality in both raw and heat processing meat materials

Song Zhou[1, †], Guowei Zhong[2, †], Hanxiao Zhou[1], Xiaoxia Zhang[3], Xiaoqun Zeng[1], Zhen Wu[1], Daodong Pan[1,*], Jun He[1,*], Zhendong Cai[1,*]

Supplementary Material

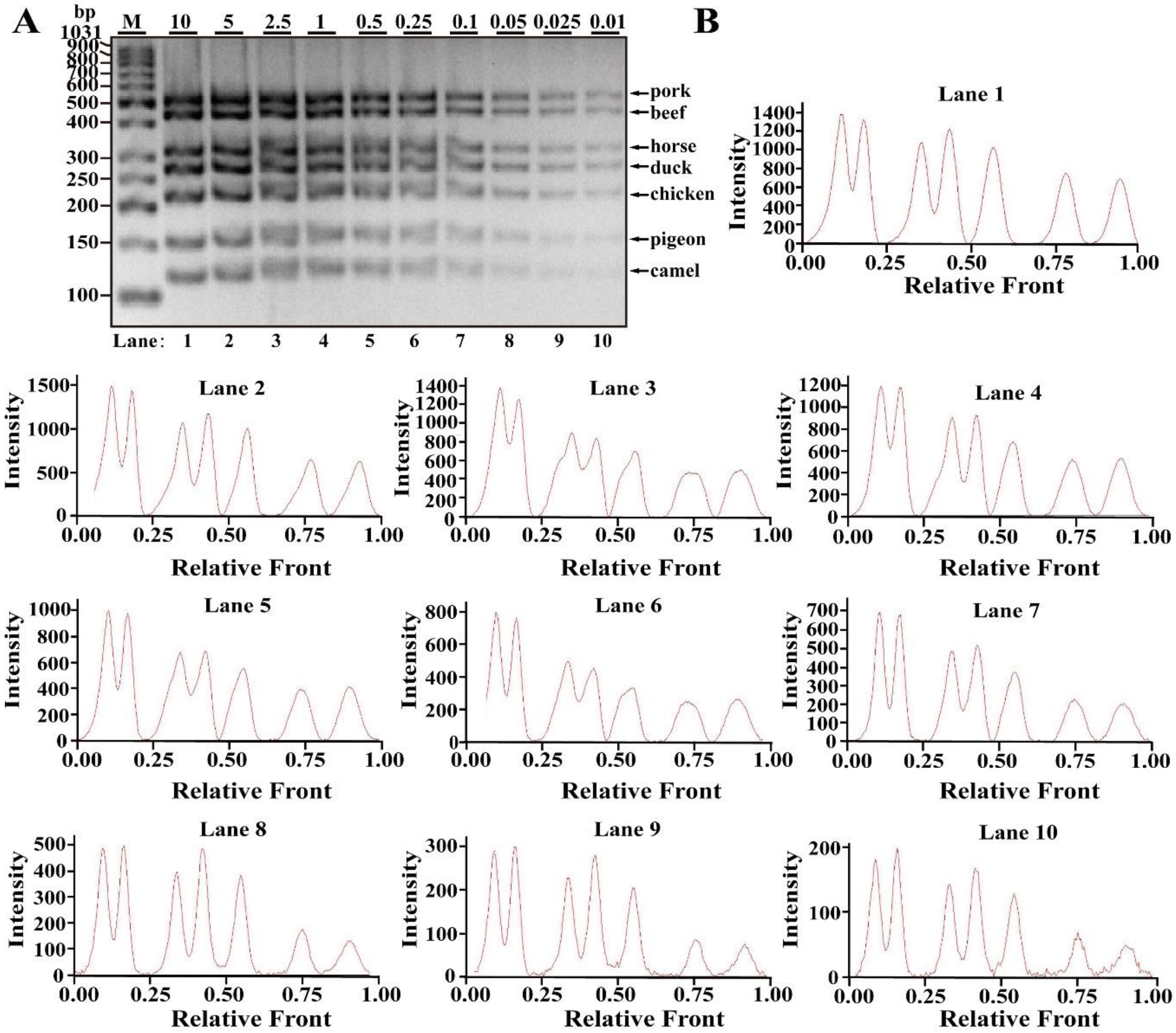

Supplementary Figure 1. Validation of the reproducibility of multiplex PCR assay in boiled meat samples. (A) Gel image of PCR fragments amplified by multiplex PCR using premixed DNA templates of seven species (10, 5, 2.5, 1, 0.5, 0.25, 0.1, 0.05, 0.025 and 0.01 ng) with species-specific primers of seven meat species in a single PCR reaction. (B) The corresponding electropherogram of gel image represented pork, beef, horse, duck, chicken, pigeon and camel in each lane. Lanes 1–10 are presented with labels (10, 5, 2.5, 1, 0.5, 0.25, 0.1, 0.05, 0.025 and 0.01) in (A). The value of number at the horizontal line means the relative position of peaks distant from the top of agarose gel. The value of number at the vertical line means the fluorescent intensity of DNA-bound dyes using 4S GelRed Nucleic Acid Stain. Lane M is ladder DNA.

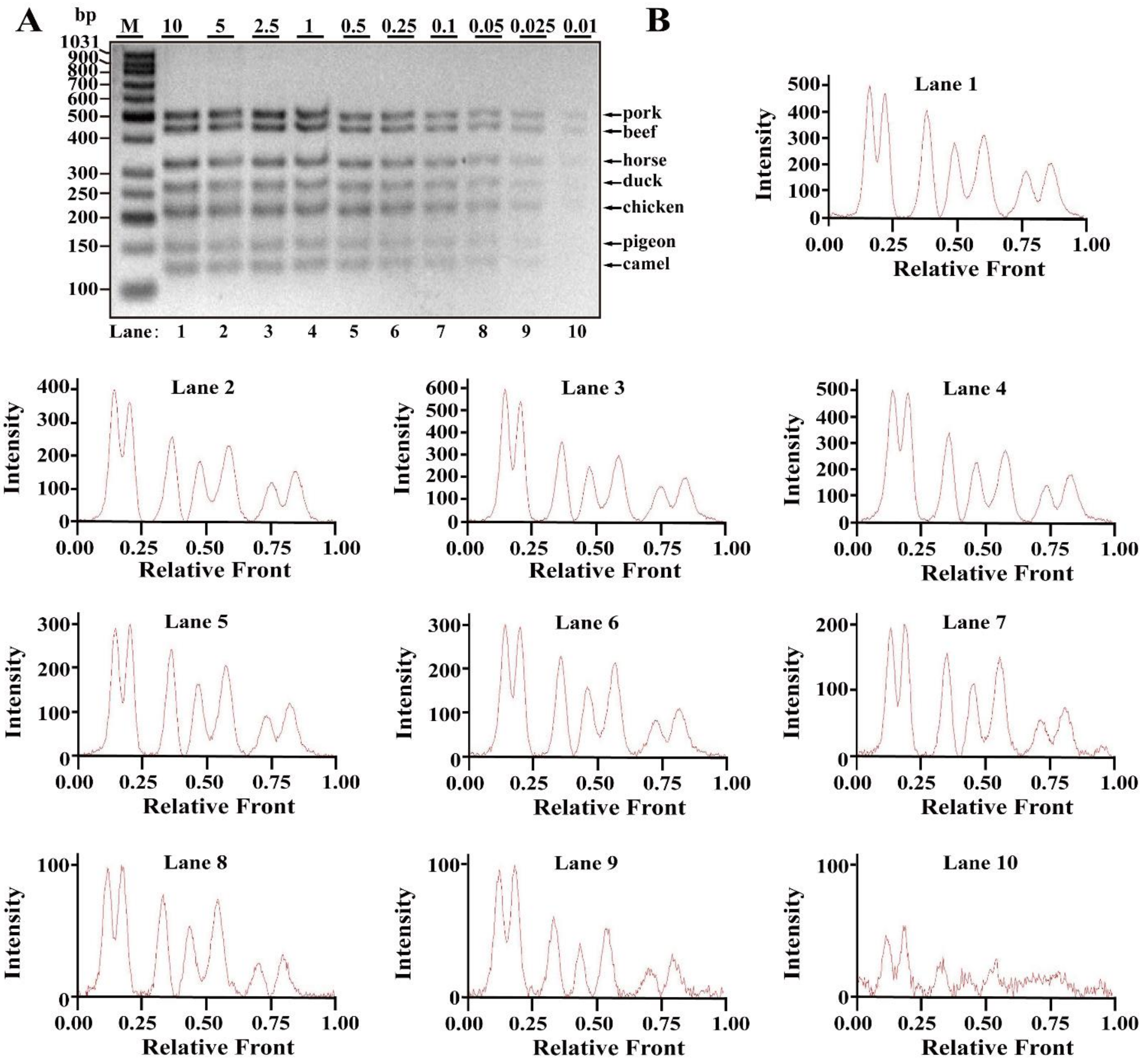

Supplementary Figure 2. Validation of the reproducibility of multiplex PCR assay in microwave-cooked meat samples. (A) Gel image of PCR fragments amplified by multiplex PCR using premixed DNA templates of seven species (10, 5, 2.5, 1, 0.5, 0.25, 0.1, 0.05, 0.025 and 0.01 ng) with species-specific primers of seven meat species in a single PCR reaction. (B) The corresponding electropherogram of gel image represented pork, beef, horse, duck, chicken, pigeon and camel in each lane. Lanes 1–10 are presented with labels (10, 5, 2.5, 1, 0.5, 0.25, 0.1, 0.05, 0.025 and 0.01) in (A). The value of number at the horizontal line means the relative position of peaks distant from the top of agarose gel. The value of number at the vertical line means the fluorescent intensity of DNA-bound dyes using 4S GelRed Nucleic Acid Stain. Lane M is ladder DNA.

Supplementary Table 1. Comparative analysis of multiplex PCR assays for the identification of species origin of meat.

Multiplex PCR type	Species number	Detection items	Detection Limit	Detection method	Reference or Source
Septuple	7	pig, beef, horse, duck, chicken, pigeon, camel	0.01-0.025 ng DNA	Gel	This study
Multiplex	2	cattle, horse	0.05 ng DNA	Gel	(1)
Tetraplex	3	pig, cattle, fish	0.001–0.1 ng DNA	Gel	(2)
Quadruplex	4	chicken, mutton, beef, pork	16 pg DNA, 0.01% of each species	Gel	(3)
Pentaplex	5	dog, duck, buffalo, goat, sheep	0.1-0.32 ng DNA	Gel	(4)
Multiplex	3	chicken, turkey, duck	1 pg for each species	Gel	(5)
Multiplex (two-tube)	14	cattle, donkey, Canidae (dog, fox, raccoon-dog), deer and horse; pig, Ovis (sheep, goat), poultry (chicken, duck), cat, mouse	0.02-0.2 ng DNA	Chip	(6)
Multiplex	6	goat, chicken, cattle, sheep, pig, horse	0.25 ng	Gel	(7)
Multiplex	3	chicken, duck, goose	0.05 ng DNA or 1% for each species	Gel	(8)
Multiplex	4	chicken, duck, pork, beef	0.05% for each species	Gel	(9)
Multiplex	5	sheep/goat, bovine, chicken, duck, pig	0.5 ng DNA	Gel	(10)
Multiplex	4	buffalo, cattle, pork, duck	1 pg DNA, 0.1% for each species	Gel	(11)
Multiplex (two-tube)	10	beef, sheep, pork, chicken, turkey; cat, dog, mouse, rat, human	30 pg DNA	Gel	(12)
Multiplex	6	mutton, pork, duck, chicken, horse, cat	9.1% of each species	Gel	(13)
Octuplex	8	dog, chicken, cattle, pig, horse, donkey, fox, and rabbit	0.05 ng/μL DNA	Gel	(14)
Septuple PCR	7	turkey, goose, pig, sheep, beef, chicken, duck	0.01-0.05 ng DNA	Gel	(15)
Hexaplex	6	horse, soybean, sheep, poultry, pork, cow	0.01% for each species	Gel	(16)

Chip, microchip electrophoresis; Gel, agarose gel electrophoresis

1. Wang WJ, Liu JJ, Zhang QD, Zhou X, Liu B. Multiplex PCR assay for identification and quantification of bovine and equine in minced meats using novel specific nuclear DNA sequences. *Food Control* (2019) 105:29-37. doi: 10.1016/j.foodcont.2019.05.016.

2. Sultana S, Hossain MAM, Zaidul ISM, Ali ME. Multiplex PCR to discriminate bovine, porcine, and fish DNA in gelatin and confectionery products. *Lwt-Food Sci Technol* (2018) 92:169-76. doi: 10.1016/j.lwt.2018.02.019.

3. Balakrishna K, Sreerohini S, Parida M. Ready-to-use single tube quadruplex PCR for differential identification of mutton, chicken, pork and beef in processed meat samples. *Food Addit Contam A* (2019) 36:1435-44. doi: 10.1080/19440049.2019.1633477.

4. Thanakiatkrai P, Dechnakarin J, Ngasaman R, Kitpipit T. Direct pentaplex PCR assay: An adjunct panel for meat species identification in Asian food products. *Food Chem* (2019) 271:767-72. doi: 10.1016/j.foodchem.2018.07.143.

5. Kim MJ, Yoo I, Yang SM, Suh SM, Kim HY. Development and validation of a multiplex PCR assay for simultaneous detection of chicken, turkey and duck in processed meat products. *Int J Food Sci Tech* (2018) 53:2673-9. doi: 10.1111/ijfs.13876.

6. Li JC, Li JP, Xu SG, Xiong SY, Yang JN, Chen X, et al. A rapid and reliable multiplex PCR assay for simultaneous detection of fourteen animal species in two tubes. *Food Chem* (2019) 295:395-402. doi: 10.1016/j.foodchem.2019.05.112.

7. Matsunaga T, Chikuni K, Tanabe R, Muroya S, Shibata K, Yamada J, et al. A quick and simple method for the identification of meat species and meat products by PCR assay. *Meat Sci* (1999) 51:143-8. doi: 10.1016/S0309-1740(98)00112-0.

8. Hou B, Meng XR, Zhang LY, Guo JY, Li SW, Jin H. Development of a sensitive and specific multiplex PCR method for the simultaneous detection of chicken, duck and goose DNA in meat products. *Meat Sci* (2015) 101:90-4. doi: 10.1016/j.meatsci.2014.11.007.

9. Qin PZ, Qu W, Xu JG, Qiao DQ, Yao L, Xue F, et al. A sensitive multiplex PCR protocol for simultaneous detection of chicken, duck, and pork in beef samples. *J Food Sci Tech Mys* (2019) 56:1266-74. doi: 10.1007/s13197-019-03591-2.

10. Wang WJ, Wang XK, Zhang QD, Liu ZH, Zhou X, Liu B. A multiplex PCR method for detection of five animal species in processed meat products using novel species-specific nuclear DNA sequences. *Eur Food Res Technol* (2020) 246:1351-60. doi: 10.1007/s00217-020-03494-z.

11. Wang LP, Hang XR, Geng RQ. Molecular detection of adulteration in commercial buffalo meat products by multiplex PCR assay. *Food Sci Tech-Brazil* (2019) 39:344-8. doi: 10.1590/fst.28717.

12. Prusakova OV, Glukhova XA, Afanas'eva GV, Trizna YA, Nazarova LF, Beletsky IP. A simple and sensitive two-tube multiplex PCR assay for simultaneous detection of ten meat species. *Meat Sci* (2018) 137:34-40. doi: 10.1016/j.meatsci.2017.10.017.

13. Xu J, Zhao W, Zhu MR, Wen YJ, Xie T, He XQ, et al. Molecular identification of adulteration in mutton based on mitochondrial 16S rRNA gene. *Mitochondrial DNA A* (2016) 27:628-32. doi: 10.3109/19401736.2014.908377.

14. Liu WW, Tao J, Xue M, Ji JG, Zhang YH, Zhang LJ, et al. A multiplex PCR method mediated by universal primers for the identification of eight meat ingredients in food products. *Eur Food Res Technol* (2019) 245:2385-92. doi: 10.1007/s00217-019-03350-9.

15. Cai ZD, Zhou S, Liu QQ, Ma H, Yuan XY, Gao JQ, et al. A Simple and Reliable Single Tube Septuple PCR Assay for Simultaneous Identification of Seven Meat Species. *Foods* (2021) 10. doi: ARTN 1083

10.3390/foods10051083.

16. Mansoor M, Bhat, Mir, Salahuddin, Imtiyaz A, Mantoo, et al. Species-specific identification of adulteration in cooked mutton Rista (a Kashmiri Wazwan cuisine product) with beef and buffalo meat through multiplex polymerase chain reaction. (2016) 9(3): 226–30. doi: 10.14202/vetworld.2016.226-230.
